# Orexin receptors exert a neuroprotective effect in Alzheimer’s disease (AD) via heterodimerization with GPR103

**DOI:** 10.1038/srep12584

**Published:** 2015-07-30

**Authors:** Julie Davies, Jing Chen, Ryan Pink, David Carter, Nigel Saunders, Georgios Sotiriadis, Bo Bai, Yanyou Pan, David Howlett, Annette Payne, Harpal Randeva, Emmanouil Karteris

**Affiliations:** 1 Biosciences, College of Health and Life Sciences, Brunel University, Uxbridge, UB8 3PH, UK; 2 Division of Metabolic and Vascular Health, Warwick Medical School, University of Warwick, Coventry, CV4 7AL, UK; 3 Neurobiology Institute, Jining Medical University, Jining, Shandong, 272067, P.R. China; 4 Department of Biological and Medical Sciences, Oxford Brookes University, UK; 5 Centre for Systems and Synthetic Biology, Brunel University, Uxbridge UB83PH, UK; 6 Wolfson Centre for Age Related Diseases, King’s College London, London, SE11UL, UK; 7 Department of Computer Science, College of Engineering, Design and Physical Sciences, Brunel University, Uxbridge UB8 3PH, UK; 8 Aston Medical Research Institute, Aston Medical School, Aston University, Birmingham, B4 7ET, UK

## Abstract

Orexins are neuropeptides that regulate the sleep-wake cycle and feeding behaviour. QRFP is a newly discovered neuropeptide which exerts similar orexigenic activity, thus playing an important role in energy homeostasis and regulation of appetite. The exact expression and signalling characteristics and physiological actions of QRFP and its receptor GPR103 are poorly understood. Alzheimer’s disease (AD) patients experience increased nocturnal activity, excessive daytime sleepiness, and weight loss. We hypothesised therefore that orexins and QRFP might be implicated in the pathophysiology of AD. We report that the down-regulation of hippocampal orexin receptors (OXRs) and GPR103 particularly in the cornu ammonis (CA) subfield from AD patients suffering from early onset familial AD (EOFAD) and late onset familial AD (LOAD). Using an *in vitro* model we demonstrate that this downregulation is due to to Aβ-plaque formation and tau hyper-phosphorylation. Transcriptomics revealed a neuroprotective role for both orexins and QRFP. Finally we provide conclusive evidence using BRET and FRET that OXRs and GPR103 form functional hetero-dimers to exert their effects involving activation of ERK_1/2_. Pharmacological intervention directed at the orexigenic system may prove to be an attractive avenue towards the discovery of novel therapeutics for diseases such as AD and improving neuroprotective signalling pathways.

Orexins (OX) are neuropeptides which function to regulate the sleep-wake cycle, feeding behaviour, energy balance and homeostasis. They are produced from a prepro-orexin (PPO) molecule and cleaved into two isoforms: orexin-A (OXA) and orexin-B (OXB). OXA and OXB are the ligands for two G-protein coupled receptors (GPCR): orexin receptor 1 (OX1R) and orexin receptor 2 (OX2R). 50-80,000 OX producing neurons project to many areas of the brain including the lateral hypothalamus (LHA), locus coeruleus (LC), tuberomammillary nucleus (TMN), paraventricular nucleus (PVN) and raphe nuclei and from these areas regulate feeding and appetite and the sleep wake cycle through their receptors^1^,^2^,^3^,^4^. It has long been thought that there may be some involvement of the orexigenic system in AD. For example, the number of OR-A immunoreactive neurons is significantly decreased by 40% in AD patients, along with lower cerebrospinal fluid (CSF) levels compared with healthy controls^5^. Moreover, an association between mean Aβ42 CSF levels and OR-A levels has been documented suggestive of a relationship between AD pathology and orexin disturbances^6^. In a more recent study, orexin levels are positively correlated with Tau and phospho-Tau in 17 AD patients^7^.

QRFP is a newly discovered neuropeptide which exerts similar orexigenic activity including the control of feeding behaviour. It is the ligand for the GPCR; GPR103, which is expressed in the ventromedial hypothalamus and PVN and outside of the brain is expressed in the retina, testes, thyroid, pituitary and prostate. GPR103 also shares 48 and 47% protein sequence homology with OX1R and OX2R respectively^8^. It is in these tissues where it can exert other physiological functions including control of the gonadotropic axis, bone formation and adrenal steroidogenesis^9^. The exact expression and signalling characteristics and physiological actions of QRFP/GPR103 are still poorly understood.

AD is characterized by gradual and increasing loss of cognitive function and behavioural abnormalities, including memory loss, personality changes, confusion, loss of language skills, severe sleep disturbances, and weight loss^10^. The main causes are a build-up of the toxic protein amyloid-β (Aβ) and hyperphosphorylation of the microtubule stabilising protein: tau, leading to neurofibrillary tangles (NFT). These two hallmarks of disease result in neuronal damage and cell death causing associated symptoms and eventually death^10^. The physiological functions of the orexigenic system and the clinical symptoms observed in AD suggest a link between the two^11^. To date, signalling characteristics of OXRs and GPR103 in the human AD brain have not been investigated.

In this study we investigated the expression and cellular distribution of these GPCRs in the AD brain and provided evidence for novel neuroprotective effects based on transcriptomic data. Given that dimerization of GPCRs is crucial for receptor function including specificity of signal transduction, we demonstrate for first time that GPR103 forms a functional hetero-dimer with OXRs to exert potential neuroprotective effects.

## Results

### Expression and cellular distribution of OXRs and GPR103 in AD

**Figure 1:**
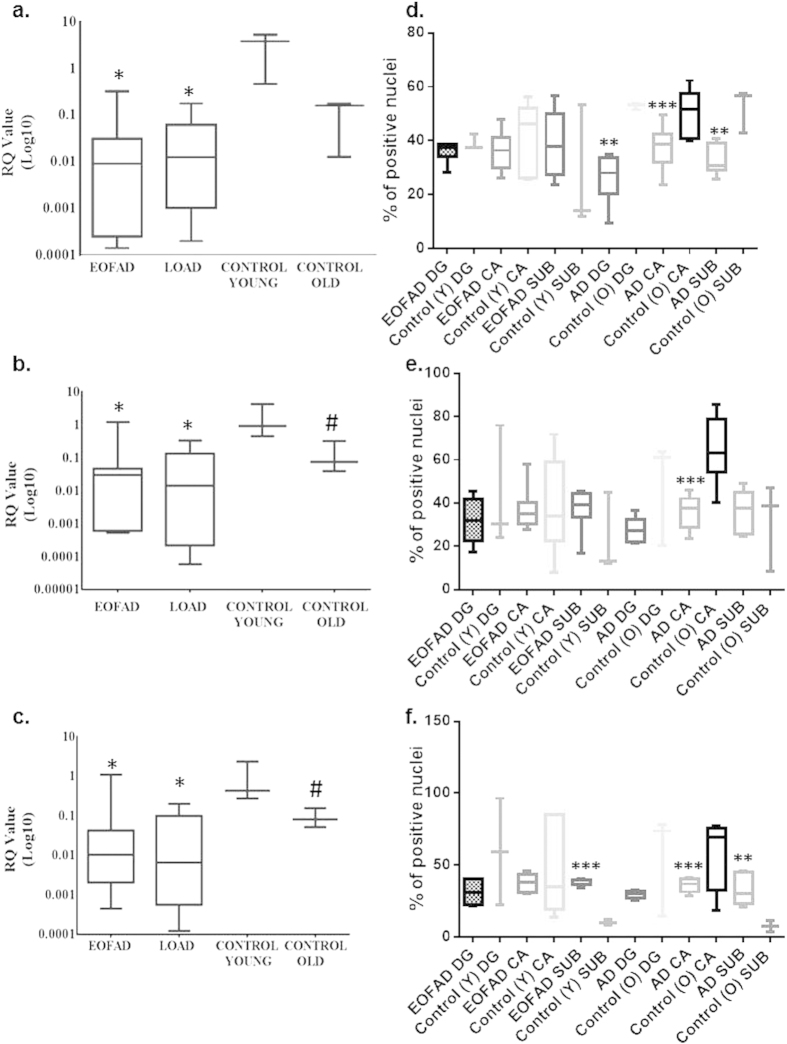
qPCR analysis of EOFAD (n = 6), LOAD (n = 6), young (n = 3) and old (=3) controls for OX1R (**a**) OX2R (**b**) and GPR103 (**c**) *p < 0.05 to control young, #p < 0.05 control young to control old. Immunohistochemical analysis of hippocampal slides for OX1R (**d**) OX2R (**e**) and GPR103 (**f**) Areas of the dentate gyrus (DG), cornu ammonis (CA) and subiculum (SUB) were counted for each patient and an average was calculated ± S.D. **p < 0.0, ****p < 0.0001 to respective controls.

In qPCR analysis, early onset familial AD (EOFAD) and late onset non-familial AD (LOAD) patients display markedly lower expression for OX1R than the control samples; with hippocampal tissue from the elderly control group exhibiting lower expression than the young control group (Fig. 1a). Expression of OX2R and GPR103 in the EOFAD and LOAD patients was significantly lower than in the young control group. OX2R and GPR103 levels for the old control group are significantly lower than the young control cohort (Fig. 1b,c). In EOFAD there was a positive correlation between OX1R, OX2R and GPR103. However for LOAD and control samples the only positive correlation was between OX2R and GPR103. To alleviate any discrepancies regarding the changes in gene expression between controls and EOFAD due to a 4 year age difference, we have recalculated the expression omitting EOFAD samples so that the average age for control is 57 years and the one for EOFAD (n = 4) is 58 years. The RQ values for OX1R, OX2R and GPR103 in control samples were: 3.2, 1.5, and 1.0, whereas the RQ values for OX1R, OX2R and GPR103 in EOFAD were: 0.08, 0.38 and 0.46 respectively.

Analyses of immunocytochemical micrographs (Suppl. Fig. 1) shown OX1R down-regulation at protein level that was primarily localized in the DG and CA subfield of the hippocampus in LOAD brains compared to healthy age-matched controls (Fig. 1d) whereas OX2R was only significantly down-regulated in the CA region of LOAD (Fig. 1e). With regards to GPR103, there was an up-regulation in the DG region for both EOFAD and LOAD compared to young and old control samples respectively, and a consistent down-regulation in the CA region of LOAD compared to old controls (Fig. 1f). Consistent with this observation, OX1R antagonist administration into CA1 of male rats is known to impair spatial learning and memory^12^,^13^.

### Amyloid plaque formation and Tau hyper-phosphorylation downregulate OXRs and GPR103

**Figure 2:**
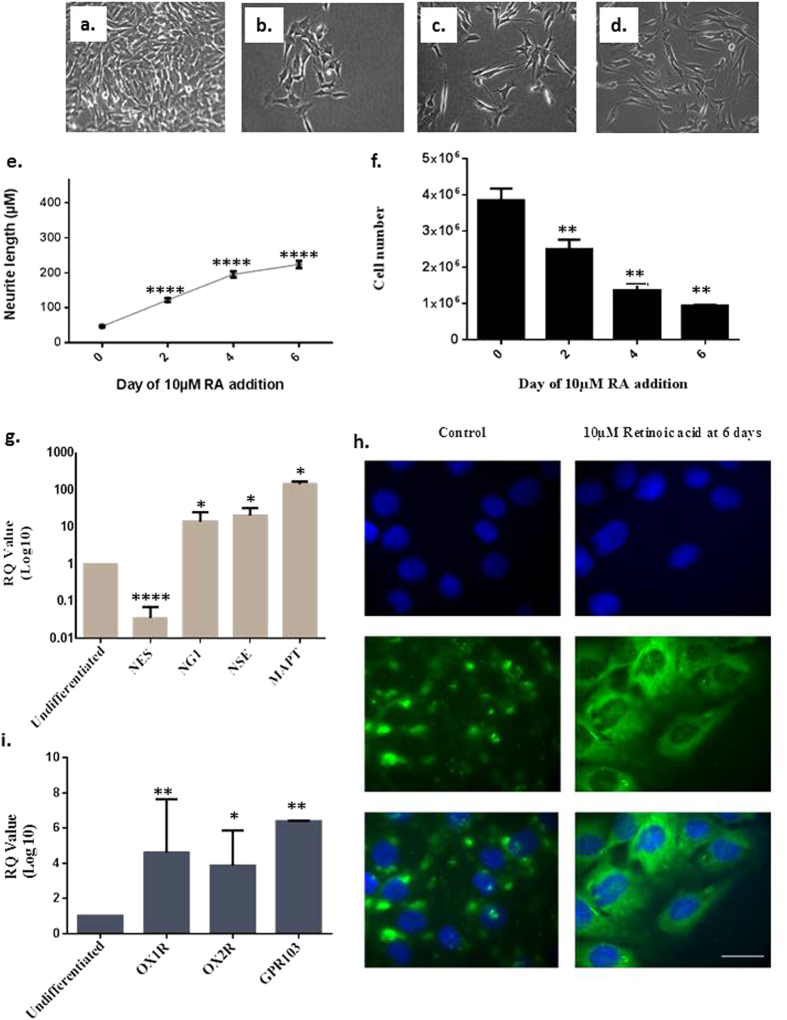
Representative images of SH-SY5Y differentiation achieved through treatment with 10 μM retinoic acid (RA). Panels: undifferentiated cells (**a**) 2-6 days 10 μM RA treatment (**b**–**d**) ×10 magnification. Average neurite length calculated as a percentage change compared to undifferentiated cells of the longest neurite extension of 24 SH-SY5Y cells over 6 days of 10 μM RA differentiation, measured by Image J ± S.D (**e**) ****p < 0.0001 compared to undifferentiated cells. SH-SY5Y proliferation was decreased upon treatment with RA up to 6 days (**f**) **p < 0.01 compared to undifferentiated cells. Gene expression changes of neuronal markers after 6 days of SH-SY5Y differentiation (**g**) *p < 0.05, ****p < 0.0001 compared to undifferentiated cells. Pan Neuronal marker immunofluorescence in undifferentiated SH-SY5Y cells and after 6 days of 10 μM RA treatment (**h**) x40 magnification. Bars = 50 μm. Gene expression changes of OX1R, OX2R and GPR103 after 6 days of SH-SY5Y differentiation compared to undifferentiated samples (**i**) *p < 0.05, **p < 0.01 to undifferentiated controls.

To investigate further the cause of the reduction of OXRs and GPR103 we established a human *in vitro* model, based on the neuronal differentiation of the neuroblastoma cell line SH-SY5Y. Neuronal phenotype was confirmed by measuring neurite length, increased expression of neuron specific enolase, microtubule-associated protein tau and neurogenin 1, and decreased Nestin (Fig. 2a–g). A pan-neuronal marker was used targeting key somatic, nuclear, dendritic and axonal proteins confirming neuronal differentiation compared to undifferentiated cells that displayed low levels of the Pan Neuronal maker expression, primarily localised to the nucleus and cytoplasm (Fig. 2h). Moreover, acquisition of neuronal phenotype was associated with increased expression of OXRs and GPR103 (Fig. 2i). This was followed by a notable increase at protein level for all 3 receptors (Suppl. Fig. 2).

**Figure 3:**
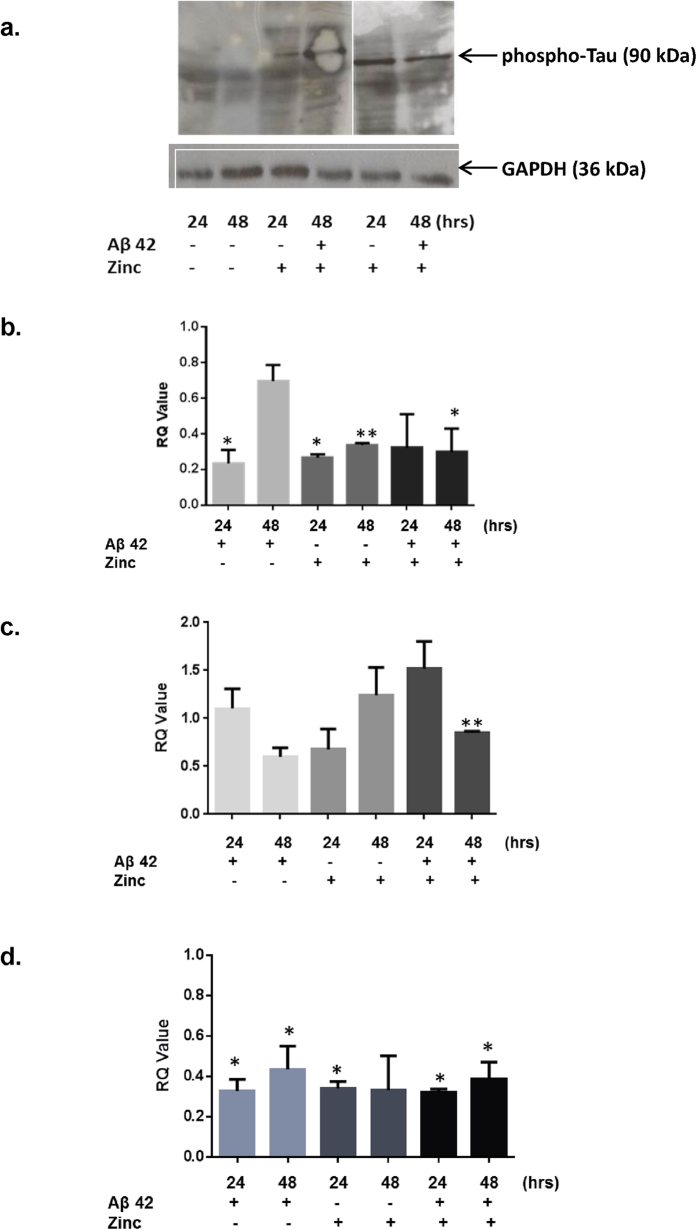
Changes in the phosphorylation status of tau following treatment with 1 μM Aβ42, 100 nM zinc sulphate or a combination at 24 and 48 hours, using GAPDH loading control of panel (**a**) Gene expression changes for OX1R (**b**) OX2R (**c**) and GPR103 (**d**) upon treatment with 1 μM Aβ42, 100 nM zinc sulphate or a combination. Results ± S.D. were obtained from 3 independent experiments. *p < 0.05, **p < 0.01 compared to no treatment controls.

Neuronal cells were treated with Aβ42 and zinc sulphate to induce amyloid deposition and tau phosphorylation respectively; mimicking an AD milieu *in vitro* as we have previously described^14^. In the case of tau phosphorylation at serine 214, it is evident that is increased following zinc sulphate treatment as there were no detectable bands in the untreated samples at this molecular weight. GAPDH was used a loading control (Fig. 3a). Tau has 6 isoforms which are expressed in the brain, and this can explain the multiple bands detected^15^.

For OX1R, OX2R and GPR103 there was a decrease in expression following Aβ42 treatment (Fig. 3b–d). Tau hyper-phosphorylation was associated with a decreased OX1R and GPR103 expression (Fig. 3b–d). At 48 hrs of Aβ42 and zinc sulphate co-treatment, all three receptors were significantly reduced in their expression. The effect however, was not additive. These findings provides evidence of how the two hallmarks of AD^16^,^17^ down-regulate these GPCRs, and indicates a mechanism for their reduced expression in clinical samples.

### Neuroprotective effects of orexins and QRFP

To elucidate further the role of these neuropeptides we performed a whole genome microarray analysis. Values which had a p-value derived from the unpaired student T test of less than 0.05 compared to the control and with a fold change compared to the control more than 1.5 or less than 0.5 were included, all others were discounted as displaying no significance. The genes generated from these stipulations were then analysed using KEGG pathway software. OXA significantly treatment of neuronal cells altered expression of 346 genes, including genes in pathways implicated in neuroprotection^18^,^19^,^20^. OXA augments NF-KB signalling that can regulate cell survival, repair, neurogenesis, learning and memory^21^ (suppl. Table 1). OXB altered expression of 715 genes involving KEGG pathways for: PI3K-Akt and Jak-STAT signalling, neuroactive-ligand receptor interaction, cytokine-cytokine receptor interaction, and AD. Up-regulation of PI3K-Akt signalling points towards a neuroprotective function of OXB (suppl. Table 2). QRFP treatment affected 2056 genes. The main KEGG pathways include: neuroactive-ligand receptor interaction, metabolic pathways, cytokine-cytokine receptor interaction, PI3K-Akt, Jak-STAT, MAPK, TNF, NF-KB and AD. QRFP regulated 16 genes involved in the PI3K-Akt and 13 MAPK signalling pathways, many of which are involved in cell survival. QRFP also regulated 13 genes involved in the MAPK pathway, and 7 voltage-dependent calcium channels that act as mediators of neurotransmitter release (suppl. Table 3). Data has been further analysed using Gene Ontology (GO) terms for Molecular Function with significance at 1.5 fold change for OXA and OXB and at 2.0 fold difference for QRFP (suppl. Table 4).

We have validated these data using qPCR. Since 69 genes were significantly altered their expression by all three peptides in a similar manner, we have decided to validate 8 genes from this cohort. The qPCR data corroborated the microarray findings, in genes with increased (CSTF2T, DAB2IP, OSBPL7, PYCRL and ZP) or decreased (GPR148, KRT23 and ZFP42) expression (Suppl. Table 5). The qPCR data demonstrated a similar degree of change as observed by microarray analyses.

### OX1R and OX2R form heterodimers with GPR103: implications in ERK_1/2_ activation

**Figure 4:**
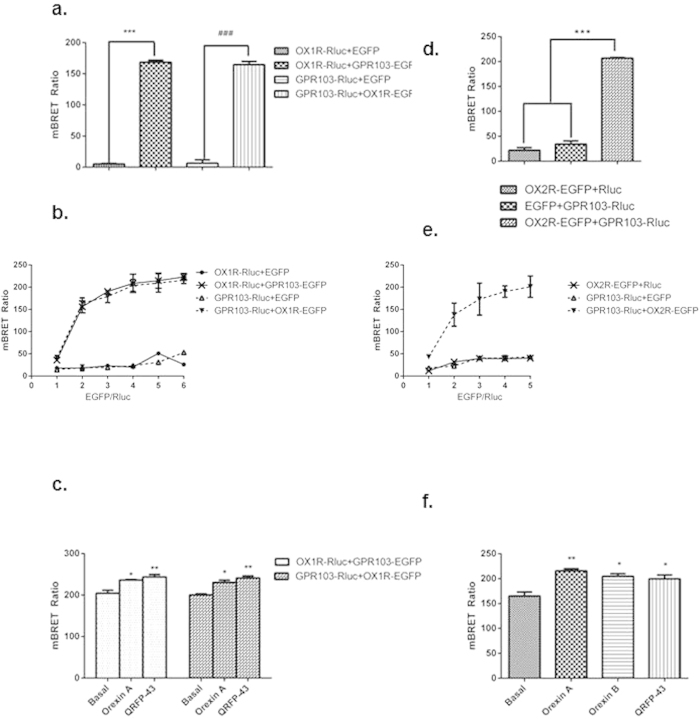
HEK293 cells were transiently transfected with 0.2 μg GPR103-Rluc or 0.2 μg OX1R -Rluc and 0.6 μg OX1R-EGFP or 0.6 μg GPR103-EGFP (**a**) ***p < 0.001 to control OX1R-Rluc + EGFP (n = 3), ^###^p < 0.001 to control GPR103-Rluc+EGFP (n = 3). HEK293 cells were co-transfected a constant amount of cDNA for GPR103-Rluc or OX1R-Rluc (0.15 μg) and increasing concentration of EGFP-tagged cDNAs. With the increasing expression (0.15–0.75 μg) of OX1R-EGFP or GPR103-EGFP, the saturation curve indicated the specific interaction between GPR103 and OX1R (**b**) GPR103-Rluc and OX1R-EGFP (1:3 and *vice versa*) were co-transfected into HEK293 cells and mBRET was measured upon treatment with OXA or QRFP (10 nM; n = 3). *p < 0.05, **p < 0.01 compared to basal unstimulated levels (**c**) HEK293 cells were transiently transfected with GPR103-Rluc, OX2R-EGFP or Rluc (all at 0.2 μg) and 0.6 μg OX2R-EGFP, or 0.6 μg EGFP (n = 3). ***p < 0.001 to controls GPR103-Rluc+EGFP and OX2R-EGFP+Rluc (**d**) HEK293 cells were co-transfected a constant amount of cDNA for GPR103-Rluc (0.15 μg) and increasing concentration (0.15–0.75 μg) of EGFP-tagged cDNAs. With the increasing expression of OX2R-EGFP and GPR103-Rluc, the saturation curve indicated a specific interaction between GPR103 and OX2R (**e**) GPR103-Rluc and OX2R-EGFP (1:3) were co-transfected into HEK293 cells and treated with OXA, OXB and QRFP (10 nM; n = 3). *p < 0.05, **p < 0.01 compared to basal levels (**f**).

**Figure 5:**
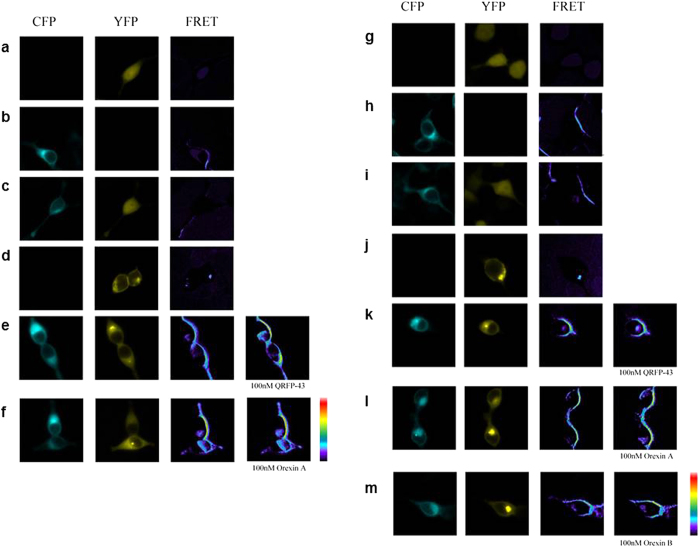
FRET imaging of constitutive GPR103 and OX1R/OX2R heteromeric interactions in living cells. GPR103-CFP and OX1R-YFP were expressed individually or co-expressed in HEK293T cells: YFP (**a**) GPR103-CFP (**b**) GPR103-CFP and YFP (**c**) OX1R-YFP (**d**) GPR103-CFP and OX1R-YFP (**e**,**f**) Upon stimulation with 100 nM QRFP-43 (**e**) or OXA (**f**) there is significant change in FRET. GPR103-CFP and OX2R-YFP were expressed individually or co-expressed in HEK293T cells: YFP (**g**) GPR103-CFP (**h**) GPR103-CFP and YFP (**i**) OX2R-YFP (**j**) GPR103-CFP and OX2R-YFP (**k**–**m**) FRET was detectable upon stimulation with QRFP (**k**) OXA (l), OXB (m; all at 100 nM). Lowest FRET intensity is in purple/black and the highest in red.

**Figure 6:**
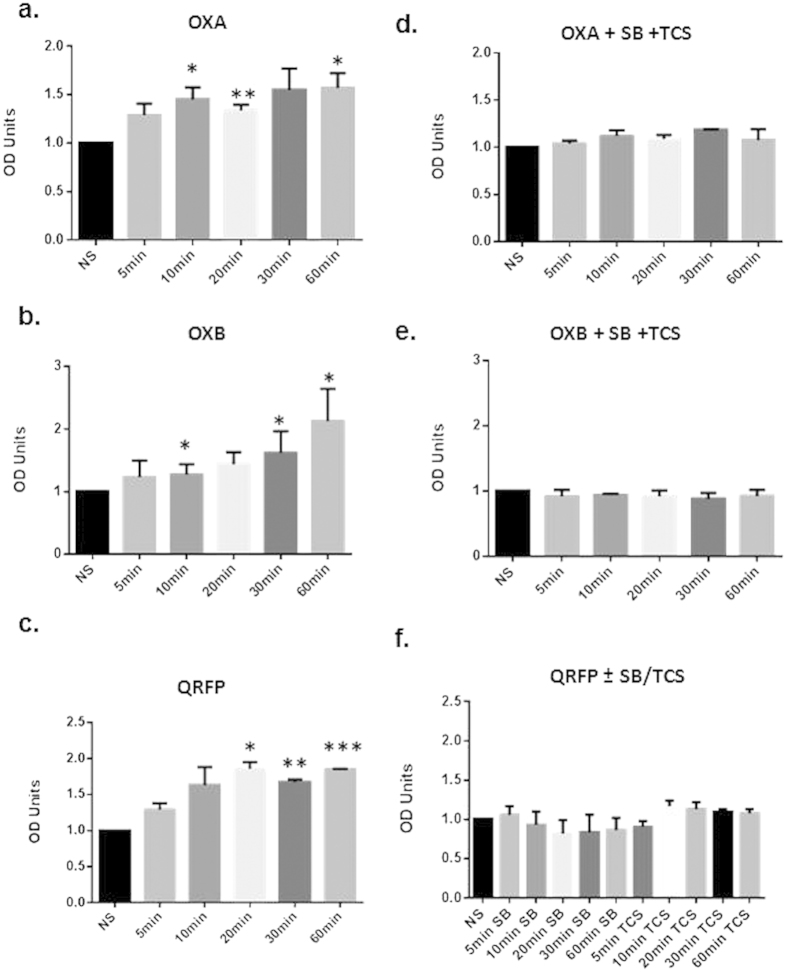
Densitometric analysis (OD Units) of p-ERK_1/2_ normalised to t-ERK_1/2_. Cells were treated with 100 nM of OXA (**a**) OXB (**b**) or QRFP (**c**) in the presence of the OXR antagonists SB (SB-334867) and TCS (TCS OX 229; (**d**-**f**). *p < 0.05, **p < 0.01, ***p < 0.001 to no supplement (NS).

Given the homology between the three GPCRs and the induction of OX1R by QRFP, it appears that there is a higher order of complexity in OXR/GPR103 signalling. We further investigated this by assessing their potential to heterodimerize. Dimerization of GPCRs is crucial for receptor function including the determination of agonist affinity, efficacy, trafficking and specificity of signal transduction, in particular G protein-coupling^22^. Using BRET (Fig. 4a–f) and FRET (Fig. 5), we provided evidence of heterodimerization between OX1R and GPR103 and OX2R and GPR103 in a constitutive and induced form. All three peptides induced ERK_1/2_ phosphorylation (Fig. 6a–c), a key molecule in the prevention of neurodegenerative processes^23^. Specific OXR antagonists SB-334867 (a selective antagonist of OX1R) and TCS OX 229 (a potent and selective antagonist of OX2R) abolished the effects of OXA and OXB (Fig. 6d–f). Treatment of neuronally-differentiated cells with both antagonists also blocked the QRFP-induced ERK_1/2_ phosphorylation, indicative of a cross-talk mechanism. Furthermore, immunofluorescent studies on neuronally differentiated SH-SY5Y cells, demonstrate co-localisation of OX1R and GPR103 as well as OX2R and GPR103, indicative of the potential of heterodimerisation (data not shown).

## Methods

### Cell culture

SH-SY5Y cells (ATCC) were cultured in 1:1 EMEM and Hams F12 (Sigma-Aldrich), with 10% FBS (50 ml), 1% non-essential amino acids, 1% 200 mM L-glutamine, 1% penicillin/streptomycin at 37 °C with 5% CO_2_. SH-SY5Y cells were neuronally-differentiated for 6 days with treatment of 10 μM retinoic acid (RA) (Sigma-Aldrich) as previously described^14^.

### Clinical samples

Anterior hippocampus with entorhinal cortex tissues from 13 AD adults (7 with early onset familial AD and 6 with late onset AD) along with 6 controls (n = 3 young, n = 3 old) were obtained from Brains for Dementia Research (BDR), University of Newcastle, UK (suppl. Table 6). BDR offers UK-based studies clinical samples and associated data obtained via BDR generic ethical approval. Informed consent was obtained from subjects. However, it is possible for a patient to take part even when they cannot themselves give consent. Agreement to take part can be given by a person in a qualifying relationship, taking into account the potential participant’s past and present wishes and feelings, beliefs and values. The assessments are a key part of BDR and patients who are at the final stages of their illness cannot be registered. The current project was approved by BDR on the basis of sufficient information about the proposed research in terms of validity, methodology, peer review process, and contribution the proposed work will make to understanding and developing treatments for dementia.

### Quantitative RT-PCR

RNA was extracted and reverse-transcribed into cDNA as previously described^24^. Relative expression of the genes of interest was assessed by quantitative PCR (Q-PCR) on an ABI Prism 7900HT Sequence detection system (Applied Biosystems) using SYBR® Green-PCR reaction mixture (Sigma Aldrich, UK). GeNorm analysis indicated that GAPDH, EIF4A2, UBC and 18s as being the most stable genes. Primers used are listed on supplementary table 7. RNAs were assayed from three independent biological replicates. The RNA levels for clinical samples were expressed as a “relative quantification” using the housekeeping gene 18s RNA (RQ) value. The “Delta Ct method” was employed for comparing relative expression results between treatments in Q-PCR^24^.

### Western Immunoblotting

Protein lysates in 1X Laemmli Buffer (Sigma-Aldrich, UK) were separated on an SDS-10% polyacrylamide gel and the proteins were transferred to a nitrocellulose membrane. The membrane was blocked in TBS containing 5% dried milk powder (w/v) and 0.1% Tween-20, for 1 hr at room temperature. After three washes with TBS-0.1% Tween-20, the nitrocellulose membranes were incubated with primary antibodies against phospho-Tau (DAKO), Aβ^42^, phospho- and total-ERK_1/2_ and GAPDH (Cell Signalling Technology). The primary antisera were used at a 1:1000 dilution overnight at 4 °C. The membranes were washed thoroughly for 30 min with TBS-0.1% Tween, before incubation with the secondary HRP-conjugated immunoglobulin (1:2000) for 1 hr at room temperature and further washing for 30 min with TBS-0.1% Tween-20. Antibody complexes were visualised as previously described^14^.

### Immunofluorescent analysis of SH-SY5Y cells

Neuronally differentiated SH-SY5Y cells were fixed in 4% paraformaldehyde for 10 min prior to washes in PBS and incubation with 10% bovine serum albumin (BSA) or donkey serum in PBS for 1 hr. Cells were incubated for 1 hr with OX1R, OX2R, GPR103 (Santa Cruz Biosciences) or Pan-Neuronal marker (Millipore) antibodies at a 1:100 dilution in 1% BSA/PBS. Cells were then washed with PBS prior to a further incubation with secondary antibodies as previously described (21). Images were captured using a Plan Apo Neofluar 63X NA 1.25 oil objective (Zeiss) on a Zeiss Axiovert 200 M microscope and viewed using AxioVision software.

### Immunohistochemistry (IHC) using 3,3′-diaminobenzidine (DAB) staining

Paraffin embedded slides from corresponding patients were deparaffinised in Histoclear (National Diagnostics) for three, 5 min washes and rehydrated in ethanol for 3 min each using the following gradients; Histoclear: ethanol (1:1), 100%, 95%, 70%, 50% and ddH2O. For antigen retrieval, slides were then incubated with heated sodium citrate buffer in the microwave for 20 minutes, just below boiling point. Slides were cooled in water and washed twice for 5 min in 1xTBS-0.25% triton X (Fisher Scientific). Slides were blocked for 1 hr at room temperature in 1% donkey serum. After blocking, slides were drained and OX1R, OX2R and GPR103 primary antibodies were diluted in TBS (1:75) and added to the slides that were subsequently incubated at 4 °C overnight. The following day slides were washed three times for 5 min in TBS and then incubated with 0.3% hydrogen peroxide (Fisher Scientific) in TBS for 30 minutes to prevent any endogenous hydrogen peroxide activity. After series of 5 min washes, corresponding secondary antibody was diluted in TBS, added to each slide and incubated for 1 hr at room temperature. After secondary antibody incubation, slides were washed in TBS for three, 5 min washes. DAB (VectorLabs) reagent was prepared according to manufacturer’s instructions. Slides were incubated for 2–10 min, whilst colour change was observed. After the reaction had occurred slides were washed for 5 minutes in ddh2O. Slides were then counterstained with Mayers’ hematoxylin (Fisher Scientific) for 30 seconds, which was washed off and slides were then stained with 0.1% sodium bicarbonate for 30 seconds to achieve a blue stain. Slides then dehydrated in gradients of ethanol using: 50%, 70%, 95%, 100%, Histoclear: ethanol (1:1) and Histoclear. 100μl of di-N-butyl phthalate in xylene (DPX) mounting medium (Fisher Scientific) was added to each slide then mounted onto a coverslip.

### Nimblegen microarray

SH-SY5Y cells were treated with 10 μM of RA for 6 days followed by 24 hour treatment with 100 nM of OXA, OXB or QRFP. Experiments were performed as 3 independent experiments and RNA was DNase treated and extracted using a Qiagen RNeasy miniprep kit. The extracted RNA was quality verified on a 2100 expert Agilent bioanalyzer, to confirm that all samples had an RNA yield greater than 30 μg and RIN values above 9. cDNA was created from the RNA and hybridised to a Nimblegen 12plex × 135 k gene Human transcriptome microarray. The array slide was scanned at 3 μM on an InnoScan 700 microarray scanner and converted into TIFF images using MAPIX version 5.1 software. The TIFF images were then aligned to their Nimblegen design files and converted into probe intensity values using the Nimblegen DEVA software. This data was then Loess normalised using R statistical program and then quantile normalised for array variation using DNAstar (ArrayStar Inc.).

### FRET Imaging

FRET was used to explore further the heterodimerization between NTSR1 and APJ in living cells. The fluorescence of FRET channel (raw FRET) comprises of the actual FRET and also the bleed-through from the fluorescent proteins, namely, raw FRET = FRET + (A*receptor) + (B*donor), so this FRET signal was corrected using Coefficient A and Coefficient B, which describe bleed through correcting the raw FRET image using the sensitized emission algorithm^25^. To obtain these coefficients, cells were transfected with pEYFP and pECFP individually. After obtaining these coefficients, FRET was performed. Donor plasmid pCFP-GPR103 and receptor plasmid pEYFP-OX1R were co-transfected into HEK293 cells and stimulated with QRFP or OR-A (100 nM). In addition, pCFP-GPR103 and pEYFP-OX2R were also co-transfected into HEK293 cells stimulated with QRFP, OR-A, and OR-B. After 12–24 h, FRET signal was detected as previously described^22^.

### BRET measurements

Constitutive interactions between GPR103-OX1R and GPR103-OX2R, BRET measurements were performed as previously described^22^. Briefly, HEK293 cells were transiently transfected with the following combinations: pRluc-OX1R and/or pEGFP-GPR103, pRluc-GPR103 and/or pEGFP-OX1R, pRluc-OX2R and/or pEGFP-GPR103, and pRluc-GPR103 and/or pEGFP-OX2R. Cells were stimulated with OR-a, OR-B or QRFP (Phoenix Pharmaceuticals, USA) for 15 min. Following washes, cells were re-suspended in PBS followed by the addition of 5 μM Coelenterazine h for BRET measurements using the Tristar LB941 plate reader (Berthold, Germany) with two filter settings (Rluc filter, 400 ~ 475 nm; and EGFP filter, 500 ~ 550 nm).

### Statistical analysis

All methods were carried out in “accordance” with the approved guidelines. All data sets were analysed using the Levene’s test to test for equal or unequal variance. If the variance was deemed to be unequal and the data was unpaired the Mann-Whitney-U test was performed. If variance was equal and the data was unpaired, then the unpaired students T test was performed. For paired data, if there was equal variance the paired students T test was used. For unequal variance with paired data, the Wilcoxon signed-rank test was performed.

## Discussion

In this study we provide evidence for a functional role of the OXR/GPR103 system at neuronal level. In EOFAD and LOAD all the 3 receptors’ expression was significantly reduced compared to healthy controls. The mean age for EOFAD samples was 61 years old and for the young controls 57; whereas the mean age in LOAD samples is 84 years and for old controls 85 years and although no significance was reached, there was certainly a trend for reduced expression of all receptors in LOAD samples. One of the main mechanisms through which AD can exert a neurodegenerative effect is through oxidation of the RNA^26^, and ageing leads to increased oxidative damage which can result in defects in memory^27^,^28^. So although RNA oxidation is amplified in AD due to toxic insult it also occurs as a consequence of ageing and OXRs have been shown to be decreased with normal ageing^29^,^30^. This may explain why there is reduced expression in the old control compared to the young control group. Using Aβ42 and zinc sulphate to induce an AD phenotype *in vitro* by plaque deposition and tau hyperphosphorylation respectively; resulted in a down regulation of OXRs and GPR103. Previous data suggests that in AD there is a ~40% loss of orexigenic neurons and a 14% reduction in circulating OXA in CSF^5^. This implies that a mimicking of AD through Aβ and zinc could potentially lead to a loss of orexigenic neurons as seen in the clinical samples. OX and Aβ have been shown to be linked, as an OXA infusion was shown to increase the burden of Aβ in a transgenic mouse model of AD and a dual OX receptor antagonist was shown to reduce the deposition of Aβ^31^. This suggests that the orexigenic system can be manipulated to control the burden of disease.

Analysis of the microarray data provided a number of KEGG pathways and individual genes regulated by OXA, OXB and QRFP which confer neuroprotection. Of note with OXA treatment is the induced upregulation of somatostatin receptors, *VIP*, *EDN1* and the NF-KB KEGG pathway, all of which contribute to neuroprotection^32^,^33^. OXB increases *CRHR1*, *REDD1* and *EPO* which have all been heavily implicated in AD and protection^34^,^35^,^36^. QRFP treatment led to a decrease of *c-myb* and *BIM* and up regulation of many neuroprotective genes including *PDGF-β* and *EPO*, all of which are suggestive of a neuroprotective function^37^. Further analyses revealed that there were overlapping effects. OXA and OXB exert similar effects on 122 genes, OXA and QRFP on 117, whereas OXB and QRFP on 340. Moreover, 69 genes were up- or down-regulated in a similar manner by OXA, OXB and QRFP. It is also evident that there is a wide repertoire of genes that each one of the three neuropeptides can regulate distinctly. OXRs are very “promiscuous” in terms of their G protein coupling characteristics as we have shown previously^3^,^38^. To date, very little is known about the coupling characteristics of GPR103, although emerging studies suggest pleiotropic effects on a tissue-dependent manner^39^. Therefore, it is possible that upon activation, these GPCRs can couple to different G protein α-subunits and activate distinct, non-overlapping signalling pathways. Moreover, the degree of heterodimerisation will also dictate changes in signalling and subsequent activation or suppression of different sets of genes^40^. For example, heterodimerisation of serotonin 5-HT1A and 5-HT7 receptors play a role in the development and maintenance of depression, by receptor-mediated signalling and internalization^41^.

Here we also provide conclusive evidence for a formation of functional hetero-dimers of GPR103 with OXRs. Recent studies demonstrated that GPCR dimerization is of physiological relevance. For instance, dominant negative AT2 receptor oligomers induce G-protein arrest and symptoms of neurodegeneration in transgenic mice with AD-like pathology^42^. Moreover, OXRs have been shown to form constitutive homo- and heteromeric complexes with each other and with human CB1 cannabinoid receptors^43^. Given that OXRs are not always co-expressed in the brain, GPR103 may “promiscuously” dimerise to the available OXR to initiate their action as a hetero-dimer when only one of the OXRs is available^44^. Indeed, in the clinical samples there was a positive correlation between expression of GPR103 and OX2R for all of the patient samples at mRNA level. In the microarray QRFP also resulted in increased expression of OX1R, suggesting that QRFP stimulation of neuronal cells could selectively increase OX1R to enable a cross-talk at neuronal level. Moreover, OXR antagonists blocked QRFP-induced phosphorylation of ERK_1/2_. Immunofluorescent studies on our model confirmed co-localisation of OXRs and GPR103. It is attractive to speculate that co-expression of GPR103/OXRs in the hippocampus is necessary to allow GPR103 to become fully functional and subsequently activate downstream signalling pathways. Future studies on human AD brains are needed to study the potential heterodimerisation in clinical samples.

Taken together, we would like to propose the following: in the healthy brain, GPR103 forms hetero-dimers with OXRs favouring a neuroprotective environment. In AD patients, deposition of Aβ amyloid plaques and tau hyper-phosphorylation will drive a reduction at RNA and protein level of OX1R, OX2R and GPR103 which could worsen not only symptoms of weight loss and dysregulation of the sleep wake cycle but also confer a loss of neuroprotection through signalling pathways including ERK_1/2_; thus exacerbating the symptoms of the disease.

## Additional Information

**How to cite this article**: Davies, J. *et al*. Orexin receptors exert a neuroprotective effect in Alzheimer's disease (AD) via heterodimerization with GPR103. *Sci. Rep*. **5**, 12584; doi: 10.1038/srep12584 (2015).

## Supplementary Material

Supplementary Information
