# Supplementary material for: Orexin receptors exert a neuroprotective effect in Alzheimer’s disease (AD) via heterodimerization with GPR103
Source: Sci Rep. 2015 Jul 30;5:12584. doi: 10.1038/srep12584 (PMC4519789; doi:10.1038/srep12584)
Supplement: Supplementary Information [file srep12584-s1.pdf]

Orexin receptors exert a neuroprotective effect in Alzheimer's disease (AD) via heterodimerization with GPR103

J. Davies[1], J. Chen[2,3], R. Pink[4,] D. Carter[4], N. Saunders[5], G. Sotiriadis[1], B. Bai[3], Y. Pan[3], D. Howlett[6], A. Payne[7], H. Randeva[2], E. Karteris[1]

KEGG pathway	Number of genes involved	Genes involved
Metabolic pathways	8	PTGES, NAGLU, LALBA, GCDH, FTCD, DLST, ALOX12B
Neuroactive ligand-receptor interaction	7	GALR3, GRIN1, HRH4, HTR4, PTGER1, SSTR3, VIPR1
MAPK signalling pathway	5	FGFR4, IL1B, MAP3K14, MAPT, RASGRP1
NF-KB signalling pathway	5	BTK, IL1B, MAP3K14, SYK, TNFAIP3
TNF signalling pathway	4	EDN1, IL1B, MAP3K14, TNFAIP3
Osteoclast differentiation	4	BTK, IL1B, MAP3K14, SYK
PI3K-Akt signalling pathway	4	FGFR4, GNG13, SYK, TSC1
Cytokine-cytokine receptor interaction	4	CCR8, IL1B, IL8RA, TNFRSF19
Ras signalling pathway	4	FGFR4, GNG13, GRIN1, RASGRP1
Glutamatergic synapse	3	DLGAP1, GNG13, GRIN1
Chemokine signalling pathway	3	CCR8, GNG13, IL8RA
Calcium signalling pathway	3	GRIN1, HTR4, PTGER1
Alzheimer's disease	3	GRIN1, IL1B, MAPT
Serotonergic synapse	3	ALOX12B, GNG13, HTR4

Supplementary Table 1: KEGG pathways associated with OXA treatment in SH-SY5Y

KEGG pathway	Number of genes involved	Genes involved
Metabolic pathways	16	THTPA, SDS, PTGES2, PPOX, PLCG2, PIGO, IVD, GPT, FUK, CSAD, B3GAT3, AUH, ALG10, AKR1A1, AGXT, ACAA1
PI3K-Akt signalling pathway	10	CCND3, CSF3R, DDIT4, EPO, FGFR4, GNG13, ITGA3, JAK2, LAMB2, SYK
Neuroactive ligand-receptor interaction	10	AVPR1B, CHRND, CRHR1, DRD3, GRM4, HTR4, NMUR1, P2RY6, PTGER1, SSTR3
Pathways in cancer	8	CSF3R, HHIP, ITGA3, KLK3, LAMB2, PLCG2, STAT5A, WNT11
Jak-STAT signalling pathway	7	CCND3, CSF2RB, CSF3R, EPO, IL22RA1, JAK2, STAT5A
Biosynthesis of secondary metabolites	6	ACAA1, AGXT, AKR1A1, DHDDS, PPOX, SDS
Cytokine-cytokine receptor interaction	6	CCR10, CSF2RB, CSF3R, EPO, IL22RA1, TNFSF14
Calcium signalling pathway	5	AVPR1B, CACNA1C, HTR4, PLCG2, PTGER1
Vascular smooth muscle contraction	5	ADCY6, AVPR1B, CACNA1C, PPP1R12C, PRKCD
Hematopoietic cell lineage	5	CD2, CD8B, CSF3R, EPO, ITGA3
Glutamatergic synapse	5	ADCY6, CACNA1C, GNG13, GRM4, SLC1A6
Peroxisome	5	ABCD4, ACAA1, AGXT, PECR, SLC27A2
Cholinergic synapse	5	ADCY6, CACNA1C, GNG13, JAK2, KCNQ2
Ras signaling pathway	4	FGFR4, GNG13, PLCG2, RASA4
Dilated cardiomyopathy	4	ADCY6, CACNA1C, ITGA3, LMNA

Chemokine signalling pathway	4	ADCY6, CCR10, GNG13, JAK2
Focal adhesion	4	CCND3, ITGA3, LAMB2, PPP1R12C
Cell adhesion molecules	4	CD2, CD8B, LRRC4, PTPRF
HIF-1 signalling pathway	3	EPO, PLCG2,TF
Alzheimer's disease	1	CACNA1C

Supplementary Table 2: KEGG pathways associated with OXB treatment in SH-SY5Y

KEGG pathway	Number of genes involved	Genes involved
**Neuroactive ligand-receptor interaction**	26	ADRA1A, ADRA1B, AVPR2, CHRM5, CHRND, DRD3, GALR3, GCGR, GHRHR, GHSR, GLRA1, GRM4, GZMA, HCRTR1, HRH1, HRH3, MC3R, OPRM1, P2RX1, P2RX3, P2RX7, P2RY11, SSTR3, SSTR4, TBXA2R, TSHR
**Metabolic pathways**	24	ABO, ACAA1, ALG10, ALOX12, ALOX12B, ALOX15, ALOX15B, CYP4A11, CYP4F8, DPYS, EHHADH, FAHD1, FLAD1, FUK, IDUA, LTC4S, MVD, NDUFS7, NNMT, PCYT2, PKLR, PLCG2, PRODH, SDS
**Cytokine-cytokine receptor interaction**	21	CCL20, CCL3, CCL4, CCL5, CCR4, CSF2, CSF2RB, CSF3, CSF3R, EPO, IL11, IL17E, IL21, IL22, IL3RA, IL6, PDGFB, TNFSF14, TNFSF15, TNFSF18, XCR1
**Calcium signalling pathway**	19	ADRA1A, ADRA1B, CACNA1A, CACNA1B, CACNA1I, CACNA1H, CHRM5, ERBB2, ERBB3, GNA14, HRH1, ITPR1, P2RX1, P2RX3, P2RX7, PLCG2, PLN, RYR3, TBXA2R
**PI3K-Akt signalling pathway**	16	BCL2L11, CCND3, CHAD, CSF3, CSF3R, EPO, FGFR4, IL3RA, IL6, INS, ITGA11, ITGB4, JAK3, LAMC3, MYB, PDGFB
**Jak-STAT signalling pathway**	14	CCND3, CSF2, CSF2RB CSF3, CSF3R, EPO, IL11, IL21, IL22, IL3RA, IL6, JAK3, SOCS5, STAT5A

**Hematopoietic cell lineage**	14	CD2, CD34, CD37, CD4, CD8A, CD8B, CSF2, CSF3, CSF3R, EPO, GP9, IL11, IL3RA, IL6
**HTLV-I infection**	14	ATF3, CCND3, CSF2, E2F1, FZD3, IL6, JAK3, MYB, NRP1, PCAF, PDGFB, STAT5A, TERT, XBP1
**Pathways in cancer**	13	ARNT, CSF3R, E2F1, ERBB2, FZD3, IL6, KLK3, LAMC3, PAX8, PDGFB, PLCG2,STAT5A, STK4
**MAPK signalling pathway**	13	CACNA1A, CACNA1B, CACNA1H, CACNA1I, CACNA2D3, CACNB2, CACNG5, FGFR4, MAP3K5, MEF2C, PDGFB, STK3, STK4
**Regulation of actin cytoskeleton**	11	CHRM5, FGD3, FGFR4, GRLF1, INS, ITGA11, ITGB4, MYL2, MYL5, MYL9, PDGFB
**Proteoglycans in cancer**	11	ERBB2, ERBB3, FZD3, GAB1, GPC3, HSPB2, HSPG2, ITPR1, NUDT16L1, PLCG2, TIMP3
**Focal adhesion**	11	CCND3, CHAD, ERBB2, GRLF1, ITGA11, ITGB4, LAMC3, MYL2, MYL5, MYL9, PDGFB
**Ras signalling pathway**	9	FGFR4, GAB1, INS, PDGFB, PLCG2, RASA4, RASAL2, REL, STK4
**Chemokine signalling pathway**	9	CCL20, CCL3, CCL4, CCL5, CCR4, FGR, HCK, JAK3, XCR1
**Maturity onset diabetes of the young**	8	FOXA2, INS, NKX2, NR5A2, PAX4, PAX6, PKLR, TCF1
**MicroRNAs in cancer**	8	BCL2L11,E2F1, ERBB2, ERBB3, FZD3, PDGFB, PLCG2, TIMP3
**Serotonergic synapse**	8	ALOX12, ALOX12B, ALOX15, LOX15B, CACNA1A, CACNA1B, ITPR1, KCNJ5
**Transcriptional misregulation in cancer**	8	CEBPE, CSF2, GZMB, IL6, MEF2C, MPO, PAX8, REL
**ECM-receptor interaction**	8	CD47, CHAD, GP6, GP9, HSPG2, ITGA11, ITGB4, LAMC3
**Salivary secretion**	7	ADRA1A, ADRA1B, AQP5, CAMP, FXYD2, ITPR1, RYR3
**Non-alcoholic fatty liver disease**	7	BCL2L11, IL6, INS, MAP3K5, NDUFS7, PKLR, XBP1
**Biosynthesis of secondary metabolites**	7	ACAA1, ACOT7, MVD, NAGK, PDSS2, PRODH, SDS
**Arachidonic acid metabolism**	7	ALOX12,ALOX12B, ALOX15, ALOX15B, CYP4A11, CYP4F8, LTC4S

**Primary immunodeficiency**	7	AIRE, BLNK, BTK, CD4, CD79A, CD8A, CD8B
**Cell adhesion molecules**	7	CD2, CD34, CD4, CD8A, CD8B, MAG, SDC3
**Natural killer cell mediated cytotoxicity**	6	CSF2, GZMB, NCR2, NCR3, PLCG2, SH3BP2
**Arrhythmogenic right ventricular cardiomyopathy**	6	CACNA2D3, CACNB2, CACNG5, ITGA11, ITGB4, LMNA
**HIF-1 signalling pathway**	6	ARNT, EPO, ERBB2, IL6, INS, PLCG2
**Protein processing in endoplasmic reticulum**	6	DNAJC1, MAP3K5, MBTPS2, PPP1R15A, SSR1, XBP1
**Complement and coagulation cascades**	6	C1QA, C2, F3, PROC, SERPINF2, THBD
**Dopaminergic synapse**	6	CACNA1A, CACNA1B, DRD3, ITPR1, KCNJ5, PPP1R1B
**Inflammatory mediator regulation of TRP channels**	6	ALOX12,HRH1, ITPR1, PLCG2, PRKCD, TRPV4
**Glutamatergic synapse**	6	CACNA1A, GRM4, ITPR1, SLC1A1, SLC1A6, SLC1A7
**TNF signalling pathway**	6	CCL20, CCL5, CSF2, IL6, MAP3K5, MMP14
**Rheumatoid arthritis**	6	CCL20, CCL3, CCL5, CSF2, IL11, IL6
**ABC transporters**	6	ABCA1, ABCA5, ABCB9, ABCD4, ABCG1, ABCG2
**Cardiac muscle contraction**	6	CACNA2D3, CACNB2, CACNG5, FXYD2, MYL2, MYL3
**Cholinergic synapse**	6	CACNA1A, CACNA1B, CHRM5, ITPR1, KCNQ4, KCNJ4
**NF-kappa B signalling pathway**	5	BLNK, BTK,CCL4,PLCG2, TNFSF14
**Alzheimer's disease**	3	ITPR1, NDUFS7, RYR3

Supplementary Table 3: KEGG pathways associated with QRFP treatment in SH-SY5Y

**Molecular Functions (GOTERMS-OXA treatment)**	**Gene Count in pathway**	**% of total in pathway**	**p-value**
solute:hydrogen symporter activity	4	0.9	1.50E-02
solute:cation symporter activity	7	1.6	1.50E-02
symporter activity	8	1.8	2.70E-02
antigen binding	5	1.1	3.10E-02
alanine-oxo-acid transaminase activity	2	0.5	4.20E-02
L-alanine:2-oxoglutarate aminotransferase activity	2	0.5	4.20E-02
sugar:hydrogen symporter activity	3	0.7	4.40E-02
cation:sugar symporter activity	3	0.7	4.40E-02
**Molecular Functions (GOTERMS-OXB treatment)**	**Gene Count in pathway**	**% of total in pathway**	**p-value**
metal ion transmembrane transporter activity	31	2.9	1.50E-03
pyridoxal phosphate binding	10	0.9	1.60E-03
vitamin B6 binding	10	0.9	1.60E-03
enzyme activator activity	31	2.9	2.10E-03
potassium ion binding	15	1.4	5.90E-03
potassium channel activity	15	1.4	8.30E-03
di-, tri-valent inorganic cation transmembrane transporter activity	7	0.7	1.40E-02
metal ion binding	239	22.2	1.60E-02
cation binding	241	22.4	1.70E-02
protein kinase binding	15	1.4	1.90E-02
kinase binding	17	1.6	2.20E-02
calcium ion binding	62	5.8	2.20E-02
fibroblast growth factor receptor activity	3	0.3	2.40E-02

endodeoxyribonuclease activity	5	0.5	2.40E-02
cation channel activity	23	2.1	2.60E-02
ion binding	242	22.5	2.60E-02
deoxyribonuclease activity	6	0.6	2.80E-02
vitamin binding	13	1.2	3.50E-02
voltage-gated cation channel activity	14	1.3	3.90E-02
enzyme binding	37	3.4	4.40E-02
alkali metal ion binding	19	1.8	4.60E-02
transition metal ion transmembrane transporter activity	5	0.5	4.70E-02
**Molecular Functions (GOTERMS-QRFP treatment)**	**Gene Count in pathway**	**% of total in pathway**	**p-value**
peptidase activity	39	4.3	6.20E-03
enzyme activator activity	26	2.9	7.10E-03
voltage-gated calcium channel activity	6	0.7	7.10E-03
serine-type peptidase activity	16	1.8	1.10E-02
serine hydrolase activity	16	1.8	1.20E-02
calcium ion binding	56	6.2	1.20E-02
tumor necrosis factor receptor binding	5	0.6	1.30E-02
cytokine activity	17	1.9	1.30E-02
peptidase activity, acting on L-amino acid peptides	36	4	1.50E-02
serine-type endopeptidase activity	14	1.5	1.60E-02
metal ion transmembrane transporter activity	24	2.6	1.80E-02
cation channel activity	21	2.3	1.80E-02
extracellular matrix constituent, lubricant activity	3	0.3	2.60E-02
chemoattractant activity	3	0.3	2.60E-02

gated channel activity	22	2.4	2.90E-02
extracellular matrix structural constituent	9	1	3.30E-02
transmembrane receptor protein phosphatase activity	4	0.4	4.30E-02
tumor necrosis factor receptor superfamily binding	5	0.6	4.70E-02

Supplementary Table 4. Gene Ontology (GO) terms for Molecular Function with significance at 1.5 fold change for OXA and OXB and at 2.0 fold difference for QRFP.

Gene	OXA		OXB		QRFP	
	Microarray value	qPCR value	Microarray value	qPCR value	Microarray value	qPCR value
**CSTF2T**	2.41	1.42 ±0.25	2.07	1.64 ±0.05	2.58	1.48 ±0.17
**DAB2IP**	1.60	1.38 ±0.42	2.06	4.32 ±0.51	2.15	2.24 ±0.30
**GPR148**	0.48	0.97 ±0.52	0.43	0.39 ±0.11	0.47	0.66 ±0.12
**KRT23**	0.39	0.49 ±0.09	0.33	0.80 ±0.02	0.41	0.63 ±0.17
**OSBPL7**	1.50	1.81 ±0.15	1.58	2.02 ±1.01	1.75	2.01 ±0.73
**PYCRL**	2.02	1.39 ±0.16	1.86	1.59 ±0.48	2.30	3.03 ±0.30
**ZFP42**	0.46	0.82 ±0.04	0.48	0.77± 0.20	0.48	0.83 ±0.05
**ZP1**	2.20	1.47 ±0.19	1.76	3.24 ±0.33	3.43	3.36 ±0.69

Supplementary Table 5: Values for 8 genes that were similarly regulated by all thee neuropeptides obtained by microarray and qPCR validation upon 24 hour treatment with 100nM OXA, OXB or QRFP ±S.D.

Diagnosis	Age (years)	Sex	PM delay (hr)
EOFAD	66	F	8
EOFAD	64	M	79
EOFAD	58	M	28
EOFAD	64	M	39
EOFAD	65	M	Not provided
EOFAD	70	F	24
AD	84	F	29
AD	84	M	47
AD	83	F	12
AD	80	M	32
AD	88	F	84
AD	87	F	54
Con (young)	58	F	39
Con (young)	59	M	19
Con (young)	55	F	41
Con (old)	78	F	23
Con (old)	89	F	24
Con (old)	88	F	22

Supplementary Table 6. Clinical details of patients (diagnosis, age in years, sex, and PM delay in hrs).

Gene	Primer sequence 5’→3’	Forward/Reverse	Amplicon size (bp)
OX1R	CCTGTGCCTCCAGACTATGA<br>ACACTGCTGCATTCCATGAC	F<br>R	204
OX2R	TAGTTCCTCAGCTGCCTATC<br>CGTCCTCATGTGGTGGTTCT	F<br>R	261
GPR103	CGCCTCCCTTCCTCTACTCT<br>GAGATCGAGTCTCCCAGTGC	F<br>R	141
NSE	ATGCGACTAGGTGCAGAGGT<br>GCTCCAAGGCTTCACTGTTC	F<br>R	133
NES	AACAGCGACGGAGGTCTCTA<br>TTCTCTTGTCCGCAGACTT	F<br>R	220
NG1	ACGCCCTGTTTCATTCCTTAC<br>CCATCTATTGCCTGCTGACTAG	F<br>R	81
MAPT	TTTGGTGGTGGTTAGAGATATGC<br>CCGAGGTGCGTGAAGAAATG	F<br>R	72
CSTF2T	GCACAACCGGAATCATGTCG<br>TTCACTGGCAGCATTGTCCA	F<br>R	299
DAB2IP	AAAAGGAGGAACCCAGACGC<br>TTTCTTGAGGCGACTCGTAGG	F<br>R	135
GPR148	GCTCCCATACCTGTACCTGC<br>GTAAAGATGGCCTGGTGCCT	F<br>R	90
KRT23	CTCCCACAGCAAAGGCCATA<br>GAAGCTGTGTCCGGAGTTCA	F<br>R	130
OSBPL7	GAGCCAGGCTATGGGAACAT<br>AGATGGGCAGAAGGGCAGTG	F<br>R	154
PYCRL	TATGTCACTTTCAAGCTCTGGGT<br>ATCACTATGGCCCCTTCCTGG	F<br>R	271
ZFP42	TTACGTTTGGGAGGAGGTGG<br>ACATTTGTTTCAGCTCAGCGAT	F<br>R	229
ZP1	CTGGAGAAGGATGGGCGTTT<br>CAGAGTAGCGTCTTGTGCCA	F<br>R	90

Supplementary Table 7. List of primers used for qPCR analyses.

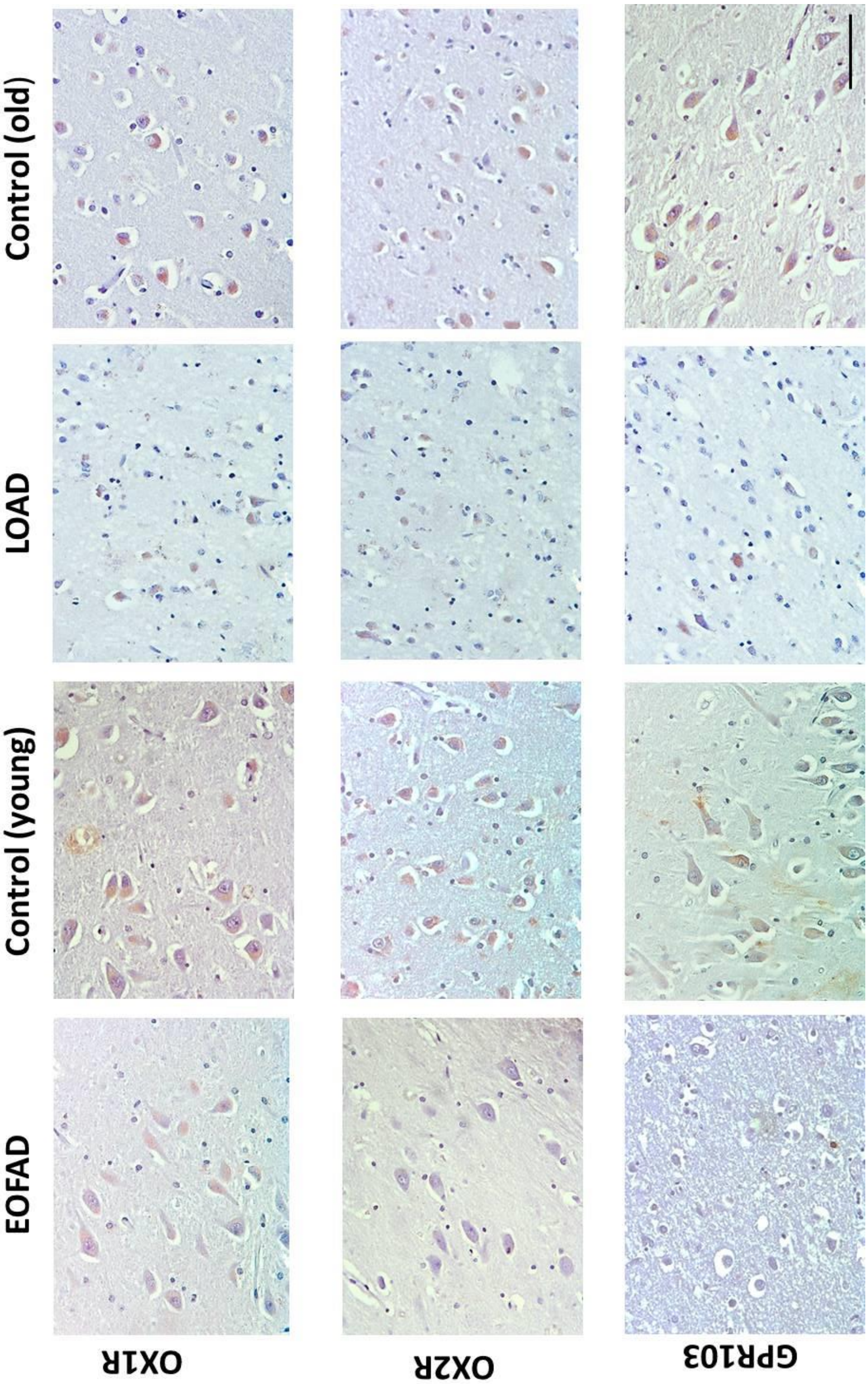

Supplementary Figure 1. Representative images of immunohistochemistry using DAB staining in EOFAD, LOAD and old and young control patients in the CA region with OX1R, OX2R or a GPR103 antibody. X40 magnification, bar = 100µM.

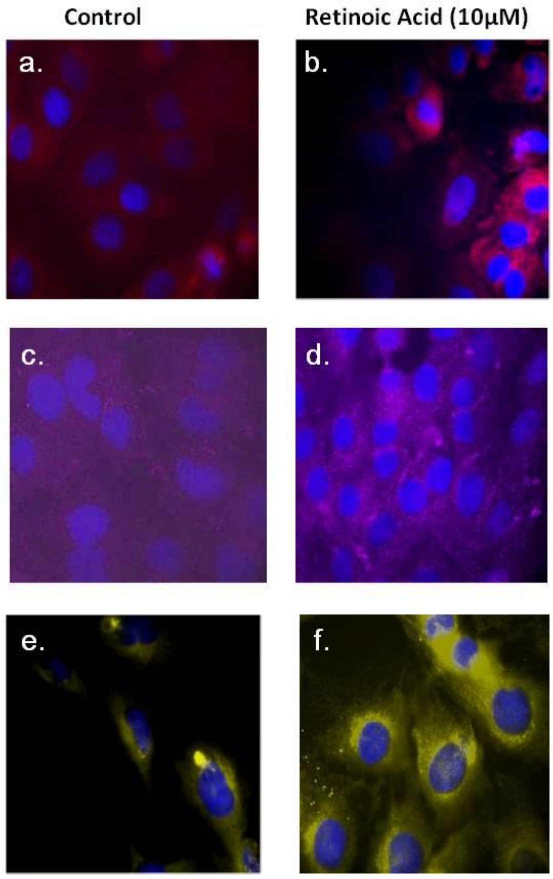

Supplementary Figure 2. Immunofluorescence using an OXIR (a, b) OX2R (c,d) and GPR103 (e,f) antibodies in SH-SY5Y cells before (a,c,e) and after differentiation (b, d, f) with retinoic acid. x40 magnification.

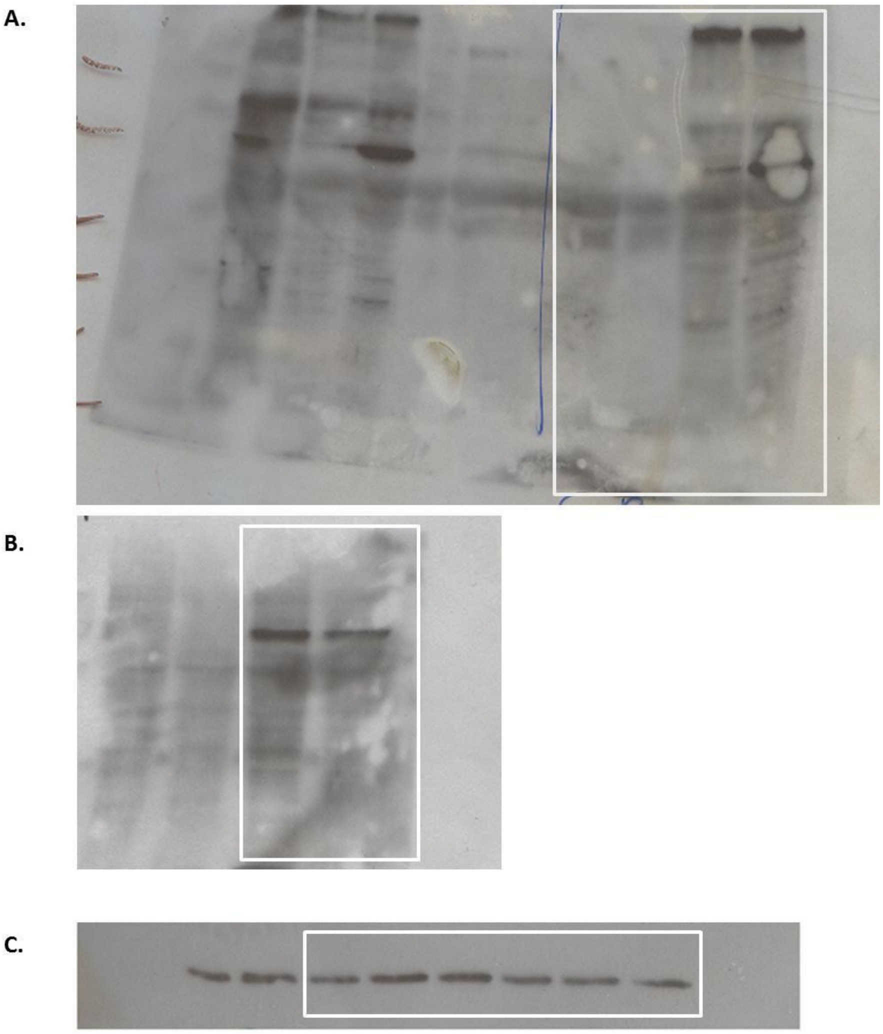

Supplementary Figure 3. Raw data for western blots for Figure 3: In white rectangle (panels A and B) images used for phospho-tau measurements. Panel C: immunoblots for GAPDH.

Supplementary Tables

Supplementary Table 1. KEGG pathways associated with OXA treatment.

Supplementary Table 2. KEGG pathways associated with OXB treatment.

Supplementary Table 3. KEGG pathways associated with QRFP treatment.

Supplementary Table 4. Gene Ontology (GO) terms for Molecular Function with significance at 1.5 fold change for OXA and OXB and at 2.0 fold difference for QRFP.

Supplementary Table 5. Values for 8 genes obtained by microarray and qPCR validation upon 24 hour treatment with 100nM OXA, OXB or QRFP ±S.D.

Supplementary Table 6. Clinical details of patients (diagnosis, age in years, sex, and PM delay in hrs).

Supplementary Table 7. List of primers used for qPCR analyses.

Supplementary Figures

Supplementary Figure 1. Representative images of immunohistochemistry using DAB staining in EOFAD, LOAD and old and young control patients in the CA region with OX1R, OX2R or a GPR103 antibody. X40 magnification, bar = 100µM.

Supplementary Figure 2. Immunofluorescence using an OXIR (a, b) OX2R (c,d) and GPR103 (e,f) antibodies in SH-SY5Y cells before (a,c,e) and after differentiation (b, d, f) with retinoic acid. x40 magnification.

Supplementary Figure 3. Raw data for western blots for Figure 3: In white rectangle (panels A and B) images used for phospho-tau measurements. Panel C: immunoblots for GAPDH.
